# Transcriptome Profile Identifies Actin as an Essential Regulator of Cardiac Myosin Binding Protein C3 Hypertrophic Cardiomyopathy in a Zebrafish Model

**DOI:** 10.3390/ijms23168840

**Published:** 2022-08-09

**Authors:** Sahar Isa Da’as, Waseem Hasan, Rola Salem, Nadine Younes, Doua Abdelrahman, Iman A. Mohamed, Arwa Aldaalis, Ramzi Temanni, Lisa Sara Mathew, Stephan Lorenz, Magdi Yacoub, Michail Nomikos, Gheyath K. Nasrallah, Khalid A. Fakhro

**Affiliations:** 1 Department of Human Genetics, Sidra Medicine, Doha P.O. Box 26999, Qatar; 2 Australian Regenerative Medicine Institute, College of Health and Life Sciences, Hamad Bin Khalifa University, Doha P.O. Box 34110, Qatar; 3 Health Center, Qatar University, Doha P.O. Box 2713, Qatar; 4 Department of Biomedical Sciences, College of Health Science, Member of QU Health, Qatar University, Doha P.O. Box 2713, Qatar; 5 Biomedical Research Center, Qatar University, Doha P.O. Box 2713, Qatar; 6 Australian Regenerative Medicine Institute, Monash University, Melbourne 3168, Australia; 7 Integrated Genomics Services, Sidra Medicine, Doha P.O. Box 26999, Qatar; 8 Imperial College London, London SW7 2BX, UK; 9 College of Medicine, Member of QU Health, Qatar University, Doha P.O. Box 2713, Qatar; 10 Weill Cornell Medical College, Doha P.O. Box 24811, Qatar

**Keywords:** hypertrophic cardiomyopathy, HCM, *c-MYBPC3*, actin, RNA seq, zebrafish knockout

## Abstract

Variants in cardiac myosin-binding protein C (cMyBP-C) are the leading cause of inherited hypertrophic cardiomyopathy (HCM), demonstrating the key role that cMyBP-C plays in the heart’s contractile machinery. To investigate the *c-MYBPC3* HCM-related cardiac impairment, we generated a zebrafish *mypbc3*-knockout model. These knockout zebrafish displayed significant morphological heart alterations related to a significant decrease in ventricular and atrial diameters at systolic and diastolic states at the larval stages. Immunofluorescence staining revealed significant hyperplasia in the mutant’s total cardiac and ventricular cardiomyocytes. Although cardiac contractility was similar to the wild-type control, the ejection fraction was significantly increased in the *mypbc3* mutants. At later stages of larval development, the mutants demonstrated an early cardiac phenotype of myocardium remodeling, concurrent cardiomyocyte hyperplasia, and increased ejection fraction as critical processes in HCM initiation to counteract the increased ventricular myocardial wall stress. The examination of zebrafish adults showed a thickened ventricular cardiac wall with reduced heart rate, swimming speed, and endurance ability in both the *mypbc3* heterozygous and homozygous groups. Furthermore, heart transcriptome profiling showed a significant downregulation of the actin-filament-based process, indicating an impaired actin cytoskeleton organization as the main dysregulating factor associated with the early ventricular cardiac hypertrophy in the zebrafish *mypbc3* HCM model.

## 1. Introduction

Hypertrophic cardiomyopathy (HCM) is one of the most common heritable cardiac conditions, with an estimated prevalence in the general population of >1:500 [1,2,3]. HCM is an archetypical single gene disorder with an autosomal dominant pattern of inheritance [4,5]. Patients usually present with fatigue, palpitations, dizziness, dyspnea, syncope, angina, and congestive heart failure. Clinical characteristics include left ventricular hypertrophy, diastolic dysfunction, outflow obstruction, and myocardial ischemia [6]. It is well-established that the clinical and genetic heterogeneity of HCM is associated with variable disease onset, prognosis, and manifestation [5,7,8]. This complexity leads to challenges in finding a curative treatment for some cases. HCM is considered as a leading cause of sudden cardiac death (SCD) in young patients and athletes [9,10,11]. To date, more than 2000 variants in at least 11 genes encoding sarcomeric proteins have been associated with HCM [10,12,13,14,15,16,17,18]. Variants in the cardiac myosin-binding protein C3 (*c-MYBPC3*) gene account for most of the genetic variants causative of HCM [12,18,19]. The MyBP-C protein is present in all slow and fast striated muscles, however, the cardiac isoform (cMyBP-C) is the abundant component of the thick filament within the cardiac sarcomere, the functional unit of the heart muscle. It possesses unique domains that play a regulatory role in the heart structure by decorating the C-zone of the A-band of the cardiac sarcomere [7,20]. Additionally, the cMyBP-C acts as a dual regulator, playing a significant role in cardiac muscle assembly and contractility [21,22,23]. Evidence suggests that cMyBP-C might act as a bridge between the cardiac sarcomere’s thick and thin filament components to modulate sarcomere contraction [24,25,26]. Specifically, the first four N-terminal domains interact with cardiac actin (thin filaments), the S1/S2 junction myosin heavy chain, and the myosin regulatory light chain [27,28] while, cMyBP-C, C-terminal domains, C7–C10, interact with myosin, the thick filament protein. Additionally, the C9–C10 domains were found to interact with titin and the C10 domain to interact with light meromyosin (LMM). Earlier studies have established that cMyBP-C acts in a phosphorylation-dependent manner as a tether between thin and thick filaments, modulating their sliding within the cardiac sarcomere [29,30,31,32]. Variants of *c-MYBPC3* causing alterations in the protein expression levels or protein function lead to severe cardiac dysfunction and structural abnormalities, particularly early-onset HCM with poor prognosis [29]. In attempts to understand the molecular and cellular switch from normal cardiac function to cardiac impairment related to the *c-MYBPC3* variants, different studies have used in vitro pathological cardiomyocyte hypertrophy cellular models and the analysis of the myocardium samples from patients [33,34,35]. However, the relevance of these approaches to human disease remains ambiguous, as these models resulted in conflicting findings and interpretations. Animal models have also played a critical role in validating *c-MYBPC3* variants, particularly those associated with severe prognosis in HCM patients. However, the pathophysiological mechanism(s) by which *c-MYBPC3* variants modulate MyBP-C function and interactions with other sarcomeric proteins remain unclear [23,36,37]. Zebrafish has emerged as an important vertebrate model for human cardiac disease modeling [38,39]. In particular, the conserved and fundamental transcriptional pathways that regulate zebrafish heart development make this model a valuable tool for studying various human cardiovascular diseases. Our study generated a *mybpc3* zebrafish knockout (KO) model to provide a concise synopsis of how *c- mybpc3* gene loss at the early stages of cardiac development leads to cardiac dysfunction results in HCM. Our *mybpc3* zebrafish KO model mimicked the human HCM presentations. The model demonstrated aberrant cardiac development and dysfunction and elucidated the pathophysiology of *mybpc3* loss through early cardiac development. Transcriptome profiling identified differentially expressed genes involved in actin organization as the key regulator of the early initiation of hypertrophic cardiomyopathy. In later developmental stages, adult mutants presented typical HCM phenotypes of a thickened ventricular cardiac wall accompanied with reduced heart rate and reduced endurance. Together, our study revealed the *mybpc3* HCM pathophysiology through the early stages of heart development.

## 2. Results

### 2.1. Human MyBP-C Is Highly Conserved in the Zebrafish Model

**Figure 1:**
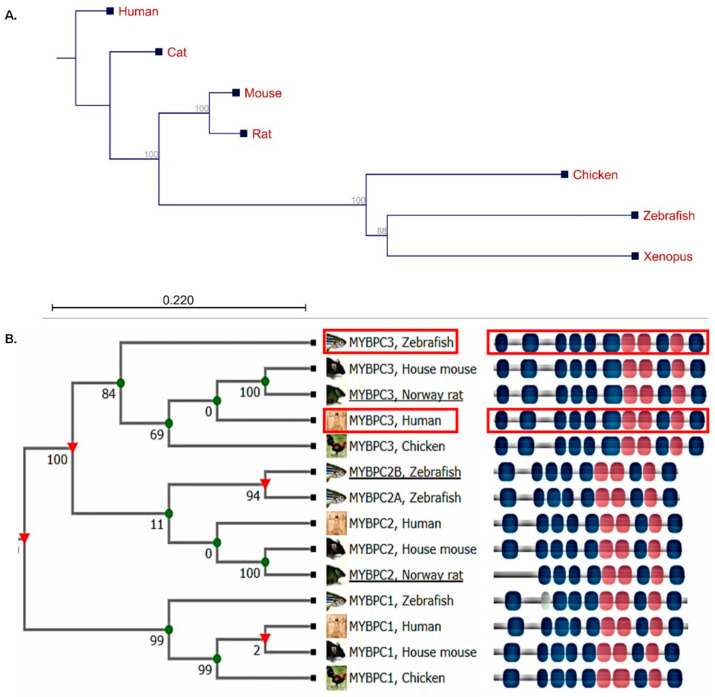
The phylogenetic tree illustrating the multiple sequence alignment analysis of the fully sequenced cardiac myosin binding protein C3 associated with hypertrophic cardiomyopathy. (**A**) The CLC sequence viewer program was used with the neighbor-joining method. The numbers for the interior branches were bootstrap percentages, and a branch length of the 0.22 site was given to the phylogenetic distances; the branching points in this tree structure were significant (>50% bootstrapping). (**B**) Human MyBP-C isoforms demonstrated evolutionary distances and conserved protein domains with the zebrafish orthologs. Red rectangle showing the human *MYBPC3* and its zebrafish ortholog *mypbc3* (TreeFam of the curated phylogenetic tree for animal gene families (http://www.treefam.org)).

The human *c-MYPBC3* (NM_000256) gene is located on chromosome 11, encoding a protein of 1274 amino acids (NP_000247), while its zebrafish ortholog *mybpc3* (NM_001044349) is located on chromosome 7, encoding a protein of 1287 amino acids (NP_001037814) [40]. A neighbor-joining phylogenetic tree for human *c-MYBPC3* orthologs was constructed to show the evolutionary distances (Figure 1A). The list of protein sequences is presented in Supplementary Materials S1. The protein sequence alignment of human and zebrafish MyBP-C proteins represented 97% coverage and shared a 62% amino acid similarity (Figure 1B). The conservation of MyBP-C suggests that zebrafish is a suitable model for studying human *c-MYBPC3* genetic variants associated with HCM.

### 2.2. Zebrafish Mybpc3 Knockout (KO) Model Generation

The injected founder generation (F0) zebrafish embryos were tested for TALEN design efficiency in targeting *mybpc3* exon 2. The specificity of the designed TALEN to target the *mybpc3* was verified via a blast run for the designed TALEN left and right sequences; this was only specific to *Danio rerio* chromosome 7, the cardiac myosin-binding protein c3 {*mybpc3*} gene of the GRCz11 genome assembly. The TALEN injection introduced a stop codon, and the BsaJI restriction enzyme site within exon 2 was lost in the *mybpc3* KO zebrafish. Genomic DNA extraction from the injected embryos showed two corresponding genomic bands, the wild-type copy that maintained the BsaJI site and the edited genomic band that lost the BsaJI site (Supplementary Figure S1). The F0 embryos were raised to adulthood and consequently outbred to wild-type zebrafish to produce the F1 generation. The F1 progeny were sequenced to identify heterozygous zebrafish and then used for in-crossing to produce an F2 generation of *mybpc3*−/−::*cmlc2*:eGFP that exhibited an intense expression of heart GFP to adulthood (Supplementary Materials S1). The F2 generation was fertile and survived into adulthood, and wild-type, *mybpc3*+/−, and *mybpc3*−/− zebrafish were born following Mendelian inheritance ratios.

### 2.3. Early Phenotype Analyses of mybpc3 Mutant Zebrafish

#### 2.3.1. Cardiac Morphology

**Figure 2:**
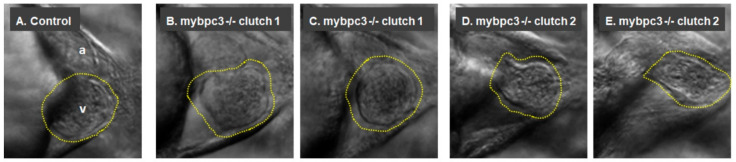
The mutant *mybpc3* displayed distinct cardiac phenotypes in the zebrafish model. Representative zebrafish heart images at the ventral view at 72 hpf. (**A**) The control showing the cardiac chambers (atrium (a) and ventricle (v)). (**B–E**) The zebrafish *mybpc3* mutant displayed distinct cardiac phenotypes. The zebrafish larvae from different clutches were mounted into 3% methylcellulose, and video recordings were taken using a Zeiss Axio-Zoom V16 stereomicroscope equipped with an Image Source Camera (60 frames per second) at 100× magnification, scale bar: 50 μm. A yellow traced line marks the zebrafish ventricle.

To characterize the role of *c-MYBPC3* on cardiac development, we examined the zebrafish *mybpc3* KO group (Figure 2). Morphological examination of the larvae at 72 h post-fertilization (hpf) showed an apparent alteration in the KO heart morphology (Figure 2B–E). Cardiac defects in KO embryos were observed compared to the wild-type zebrafish controls, which exhibited normal cardiac development. The in-crossing of the *mybpc3*−/− KO fish produced larvae that displayed the cardiac phenotype, as demonstrated by imaged hearts collected from different clutches. The *mybpc3* KO zebrafish heart displayed cardiac structural changes as it was notable that the ventricles and atria became more compact compared to the controls (Figure 2, Supplementary Videos). In addition, the ventral assessment of the cardiac chambers showed a thickened ventricle in the examined *mybpc3* KO zebrafish (Figure 2B–E, Supplementary Videos B–E) compared to the control group (Figure 2A, Supplementary Videos A,F). At the same time, the larvae with severe cardiac impairment developed cardiac edema, which severely impacted their blood flow (Supplementary Videos G–H).

#### 2.3.2. Cardiac Chambers

**Figure 3:**
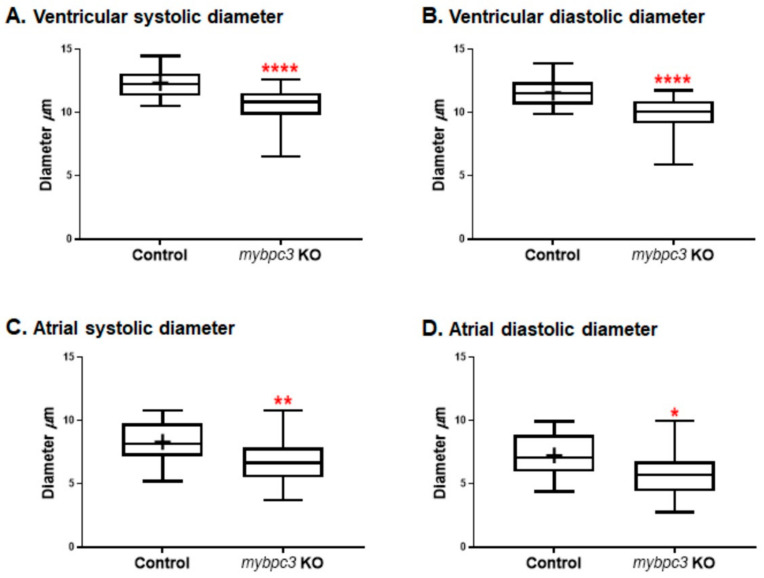
The zebrafish *mybpc3* knockout (KO) model displayed restrictive physiology of the cardiac chambers at 72 h post-fertilization (hpf). (**A,B**) Cardiac ventricular and (**C,D**) cardiac atrial analysis of the zebrafish *mybpc3* KO demonstrated by the measurement of diastolic/systolic diameters that were significantly decreased in comparison to the wild-type (control) group. The number of embryos analyzed for the control = 28 and *mybpc3* KO = 18. The larvae were mounted to image both chambers of the heart. Cardiac function analyses were represented in Box–Whisker plots and analysis using a one-way ANOVA multiple comparisons test. Values were expressed as the means ± SE. *p* values of <0.05 were considered statistically significant, a value of * *p* < 0.05, ** *p* < 0.01, and **** *p* < 0.0001.

Zebrafish larvae were imaged using a Zeiss Lumar 12 stereomicroscope to examine the cardiac morphology and function at 72 hpf. The homozygous *mybpc3* mutants displayed distinct restrictive physiology of the cardiac chambers. Gross morphological examination of the mutant heart showed smaller cardiac chambers, both the ventricle and the atrium, compared to the control group. Remarkably, the mutant myocardial wall was thicker than the control group (Supplementary Videos B–E). We also compared one-dimensional parameters of the ventricular internal chamber dimensions at end-diastole [end-diastolic diameter (EDD; largest ventricular diameter)] and end-systole [end-systolic diameter (ESD; smallest ventricular diameter)]. These measurements confirmed the substantial reductions in the ventricular EDD in the mutant compared to the control group (control EDD 12.33 ± 0.20 μm, *n* = 28; *mybpc3* KO EDD 10.53 ± 0.35 μm, *n* = 18; *p* < 0.0001). Furthermore, ventricular ESD was also significantly decreased but to a lesser extent (control ESD 11.57 ± 0.19 μm; *mybpc3* KO ESD 9.80 ± 0.34 μm; *p* < 0.0001) (Figure 2B–E and Figure 3A,B). Likewise, measurements of the atrium internal dimensions confirmed substantial reductions in the atrial EDD in the mutant group compared to the control group (control EDD 8.30 ± 0.28 μm, *n* = 28; *mybpc3* KO EDD 6.76 ± 0.45 μm, *n* = 18; *p* < 0.003). Additionally, the atrial ESD was also significantly decreased but to a lesser extent (control ESD 7.25 ± 0.30 μm; *mybpc3* KO ESD 5.8 ± 0.47 μm; *p* < 0.01) (Figure 3C,D). This suggests that the observed defective cardiac chamber development in the *mybpc3* mutant contributes to cardiac dysfunction and is attributed to the early stages of cardiac impairment.

#### 2.3.3. Cardiac Remodeling at the Cellular Level

**Figure 4:**
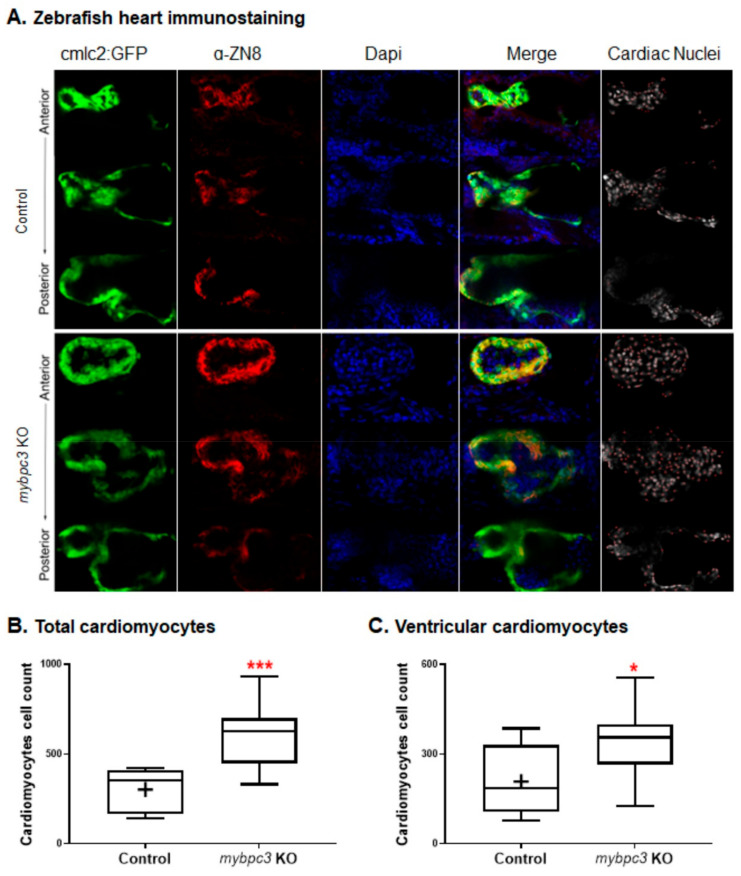
The zebrafish *mybpc3* knockout (KO) displayed cardiomyocyte hyperplasia, indicated by a significantly increased total and ventricular cardiomyocytes. (**A**) The zebrafish control Tg (*cmlc2*: GFP) and *mybpc3* KO staged at 72 hpf were stained with the ventricular cardiomyocyte cell surface marker, mouse anti-ZN-8 (red), and Dapi for their nuclei (blue). Heart chambers were scanned anterior to posterior using confocal microscopy at a Z-resolution of 3 μm, scale bar: 50 μm. The total cardiomyocytes and ventricular cardiomyocytes were quantified by scanning the whole heart; representative images demonstrated green hearts (Tg:*cmlc2*:GFP), a red ventricular cell surface, blue nucleus, merged sections, and red dots for the cell count. (**B**) Heart images were analyzed to count the total cardiomyocytes (green); *mybpc3* KO displayed a significantly increased number of total cardiomyocyte cell nuclei compared to the control. (**C**) The ventricular cardiomyocyte count (red) demonstrated that *mybpc3* KO significantly increased the cell nuclei of the ventricular cardiomyocytes compared to the control group. Datasets were analyzed by the *t*-Test using GraphPad Prism; the zebrafish group numbers analyzed were *n* = 9 for the control and *n* = 10 for *mybpc3* KO. *p* values of <0.05 were considered statistically significant, a value of * *p* < 0.05 and *** *p* < 0.001.

Since the ventricle and atrium of the *mybpc3* mutants were compact with a thicker myocardial wall compared to the control, we examined the growth of the heart chambers. Packed heart chambers can be described by increased individual cardiomyocyte size or increased cardiomyocyte number. Our *mybpc3* mutant model established the cardiac remodeling process at the cellular level. We examined the zebrafish *mybpc3* mutant embryonic hearts by immunofluorescence staining to quantify the total and ventricular cardiomyocyte cells by the overlay of green fluorescently labeled cardiomyocytes, DAPI stain, and the ZN-8 antibody (Figure 4A). The total and ventricular cardiomyocytes were significantly increased in the *mybpc3* KO compared to the controls at 72 hpf. Indeed, the count of total cardiomyocytes in the *mybpc3* mutant was 608 nuclei, *n* = 10, compared to the control (wild-type) at 302 nuclei, *n* = 9; *p* < 0.0004 (Figure 4B), and the *mybpc3* mutant ventricular cardiomyocyte nuclei count was 339 compared to the control group at 208; *p* < 0.03 (Figure 4C). These mutants developed cardiomyocyte hyperplasia in response to the genetic knockout of *mybpc3*. The zebrafish cardiac phenotype demonstrates that KO of *mybpc3* can alter cardiomyocyte biology at the early stages of heart development. The increased number of cardiomyocytes and the restrictive cardiac chamber diameters detected in *mybpc3* KO imply that myocyte hyperplasia is a physiological effect that results in overcoming the chronic myocardial wall stress in zebrafish.

#### 2.3.4. Cardiac Contractility

**Figure 5:**
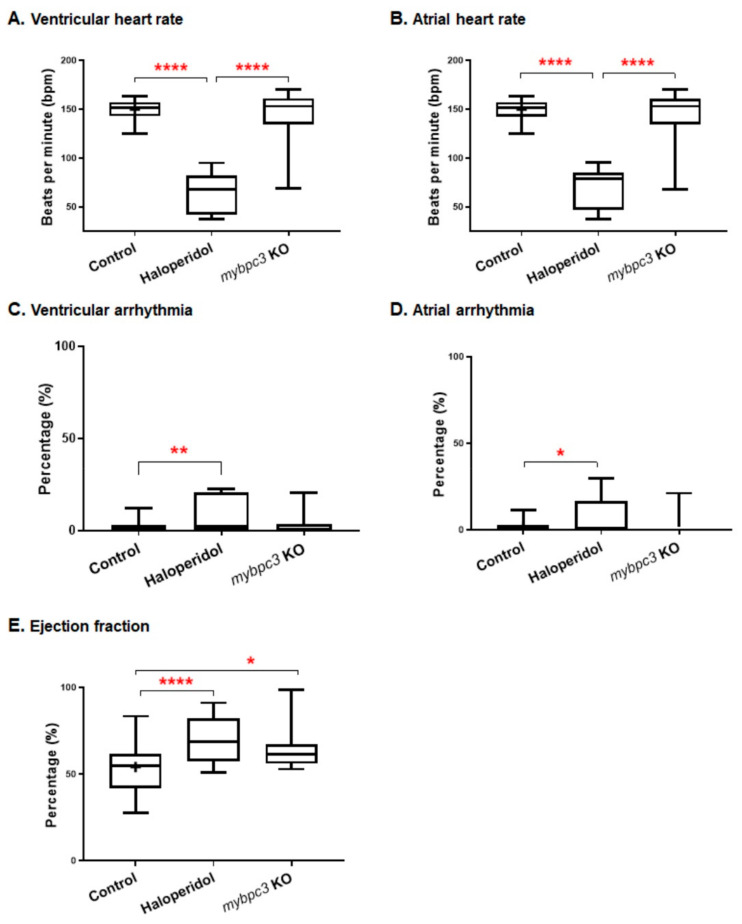
The zebrafish *mybpc3* KO cardiac function parameter analysis. The analysis of the zebrafish captured videos demonstrated a variable cardiac phenotype. (**A**,**B**) Heart rate measured at ventricular and atrial contractions. The cardiac contractility functional analysis demonstrated that *mybpc3* KO displayed a similar heart rate to the control group compared to the positive control group (haloperidol), which significantly impaired the heart rate. (**C**,**D**) Cardiac arrhythmia measurements of cardiac rhythm showed similar readings for both the *mybpc3* KO and control group compared to the haloperidol group, which produced significant ventricular and atrial arrhythmias. (**E**) Ejection fraction calculation showed that the *mybpc3* KO group exhibited a significantly increased percentage than the control group. The ejection fraction, calculated as the maximal dilatation (the diastolic diameter, DD) versus the maximal contraction (systolic diameter, SD), is measured in % as EF% = (DD − SD)/DDX100. Control EDD 12.33 ± 0.20 mm, *n* = 28; *mybpc3* KO EDD 10.53 ± 0.35 mm, *n* = 18; *p* < 0.0001). ESD was also significantly decreased, but to a lesser extent (control ESD 11.57 ± 0.19 mm; *mybpc3* KO ESD 9.80 ± 0.34 mm; *p* < 0.0001). The embryos analyzed were 28, 18, and 17 for the control, *mybpc3* KO, and haloperidol groups, respectively. One-way ANOVA using GraphPad Prism software for multiple comparisons of the *p*-value of * *p* < 0.05, ** *p* < 0.01, and **** *p* < 0.0001.

To examine the impact of *mybpc3* KO on cardiac contractility, we analyzed both the ventricular and atrial heart rates at 72 hpf. The zebrafish *mybpc3* mutants did not alter the heart beating rate compared to the control group. However, we observed a broader, more variable heart rate range within the mutant group (Figure 5A,B). The control group’s average ventricular/atrial heart rate measurements were 150/150 bpm, *n* = 28. The average was 144/144 bpm for the mutant group, *n* = 18, in contrast to the cardiotoxic drug haloperidol, which was used as the positive control and resulted in a significant reduction in the heart rate at 65/70 bpm, *n* = 17; *p* < 0.0001.

#### 2.3.5. Cardiac Rhythm

To gain further mechanistic insight into the effect of *mybpc3* knockout, we assessed the cardiac rhythm by measuring both ventricular and atrial arrhythmias. Zebrafish *mybpc3* mutants produced a non-significant increase in cardiac rhythm at 72 hpf. The control group’s ventricular/atrial rhythm measurements were at 2.2/1.8, *n* = 28, and for the *mybpc3* KO group, the percentage of beats defect was at 3.3/3.2, *n* = 18. Although we observed increased arrhythmias in the KO group; still, this difference was not significant. In contrast, embryos treated with cardiotoxic haloperidol showed arrhythmia, which was indicated by the significant increase in the percentage of beat defect at 8.4/7.1 (ventricular/atrial), *n* = 17; *p* < 0.004/*p* < 0.02 (Figure 5C,D).

#### 2.3.6. Ejection Fraction

Furthermore, we examined the functional consequences of the restrictive morphologic heart chambers phenotype in modulating the cardiac mechanistic function. The ejection fraction was calculated as an index of ventricular contractility. The *mybpc3* KO showed a cardiac functional defect; the mutant ejection fraction percentage was significantly increased at 64%, *n* = 18; *p* < 0.04, compared to the control group at 54%, *n* = 28. Similarly, cardiotoxic haloperidol treatment resulted in an increased ejection fraction percentage at 70%, *n* = 17; *p* < 0.0006 (Figure 5E). This increase in the *mybpc3* KO volumetric fraction of blood pumped out of the ventricle with each cardiac cycle relative to the maximum volume in the heart suggests ventricular cardiac dysfunction in these mutants, likewise mimicking the trend reported in patients with HCM condition [41].

### 2.4. Ventricular Hypertrophy in Adult mybpc3 Mutant Zebrafish

**Figure 6:**
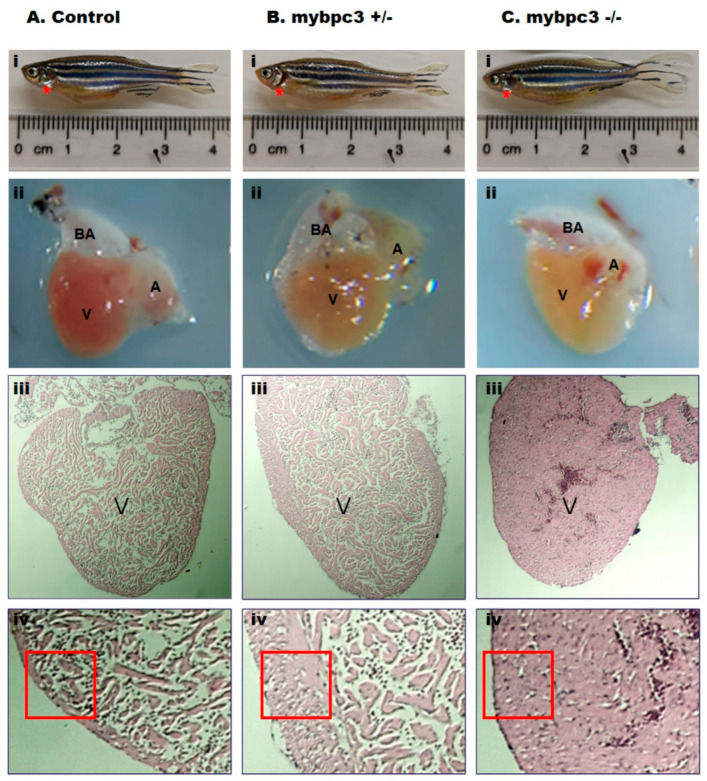
The adult zebrafish *mybpc3* mutant heart anatomy and histology. Adult age-matched zebrafish hearts were dissected (location: red star) and then compared across groups: (**A**) Control, (**B**) Heterozygous *mybpc3*+/−, and (**C**) Homozygous *mybpc3*−/− mutant. (**i**). Representation of the anatomical position of the heart in the adult zebrafish. (**ii**). The dissected heart demonstrates the two cardiac chambers, a single atrium (A), and a single ventricle (V), with an elastic, non-contractile chamber consisting of smooth muscle bulbus arteriosus (BA). (**iii**). Histological organization of the adult zebrafish ventricle, magnification 80×, scale bar: 10 μm. (**iv**). The ventricular myocardial wall is hypertrophic in the zebrafish *mybpc3* mutant model (red square) when compared to the control group subset, magnification 150×, scale bar: 10 μm.

The F2 generation was fertile and survived into adulthood. We raised three zebrafish groups: the wild-type, *mybpc3*+/−, and *mybpc3*−/−, and examined the morphology and function of the adult hearts. The heart specimen of the *mybpc3* mutant model exhibited marked ventricular hypertrophy compared to the age-matched controls (Figure 6i). The dissected *mybpc3*−/− and *mybpc3*+/− hearts were enlarged in size in comparison to the control group (Figure 6ii). Myocardial histological sections of both *mybpc3*−/− and *mybpc3*+/− demonstrated more extensive cardiomyocyte hypertrophy, myofibrillar disarray, and interstitial fibrosis than sections from the control zebrafish. H&E-stained sections of the hearts from the control wild-type zebrafish appeared normal compared to the *mybpc3*−/−, *mybpc3*+/− zebrafish sections (Figure 6iii,iv). Remarkably, the *mybpc3*−/− exhibited a more compacted and thickened ventricular wall than the *mybpc3*+/− zebrafish ventricular wall sections (Figure 6).

**Figure 7:**
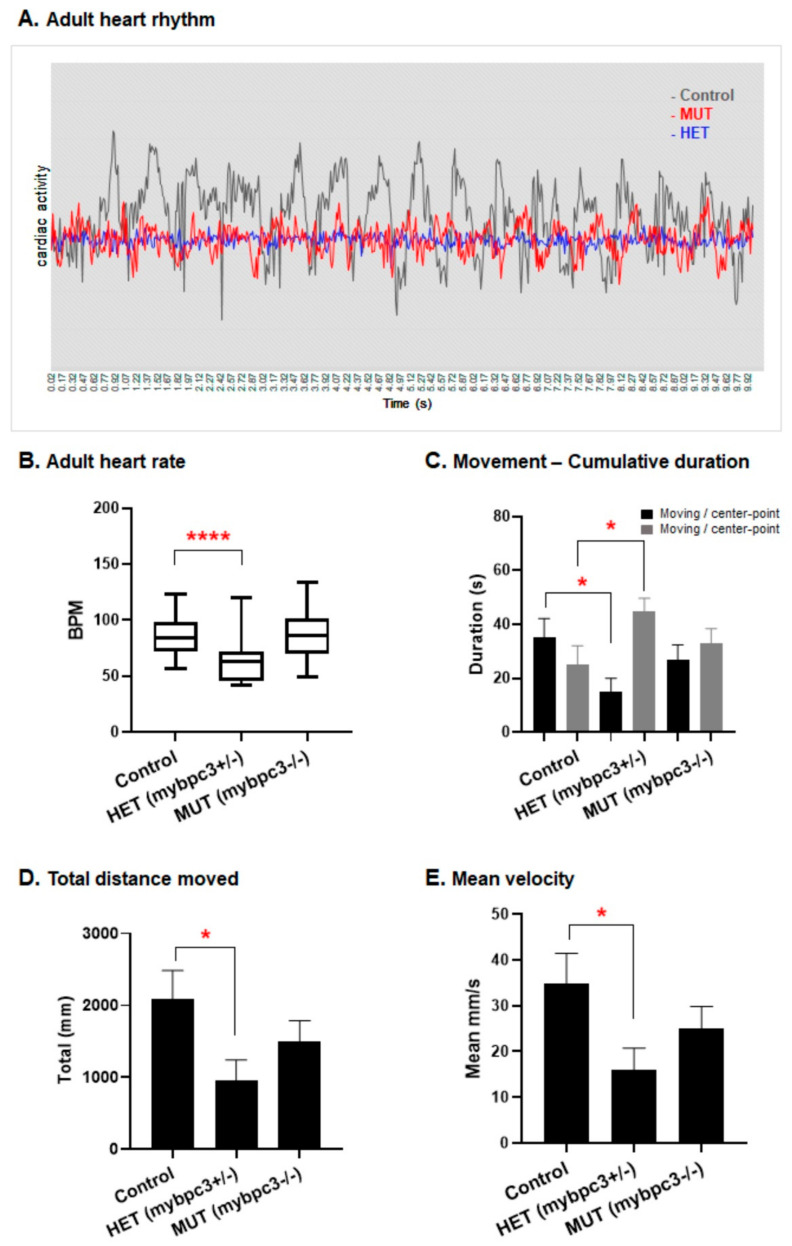
The zebrafish *mybpc3* mutant adult heart function. (**A**) Cardiac activity percentage analyzed from oscillations in the cardiac signal at the contraction-relaxation status of the ventricle, with corresponding peaks and lows at regular intervals of contractions at the correct pace over time of a total of 10 s of video recording of beating heart. Both *mybpc3*+/− and *mybpc3*+/− groups displayed decreased amplitude. (**B**) Heart rate was measured in the adult zebrafish after incubation in the combined MS-222 (Tricaine methanesulfonate) and isoflurane for minimal cardiac consequence function effects. The examined adult heterozygous *mybpc3*+/− group (*n* = 35) displayed a significant reduction in heart rate, 58.36 beats per minute (BPM), **** *p* < 0.0001 in comparison to the control (*n* = 37) at 87.61 BPM and mutant *mybpc3*−/− (*n* = 40) at 88.51 BPM. The fluorescent heart of the fish was directly imaged for 10 s using a ZEISS stereomicroscope Lumar12 supplied with an Imaging source camera (Model33UX252); data are presented as mean ± SEM. (**C**) The calculated cumulative duration of moving rate to nonmoving was lower in the *mybpc3* mutant fish. Similarly, the heterozygous *mybpc3*+/− group showed the lowest ratio decrease compared to the homozygous and control groups. (**D**,**E**) The maximum swimming speed and endurance experiments: the adult zebrafish *mybpc3* mutant demonstrated declines in the exercise capacity. The total distance and velocity decreased significantly in the *mybpc3* mutant fish. However, the heterozygous *mybpc3*+/− group showed a more significant decrease when compared to the control group. * *p* < 0.05.

To examine the cardiac function in adults, we calculated the cardiac activity percentage from oscillations in the cardiac signal at the contraction–relaxation status of the ventricle, with corresponding peaks and lows at regular intervals of contractions at the correct pace over time of a total of 10 s of video recording of the beating heart. Our analysis of the cardiac activity percentage revealed that both the *mybpc3*−/− and *mybpc3*+/− groups displayed decreased amplitude compared to the control group’s cardiac activity percentage median (Figure 7A). Remarkably, the heterozygous *mybpc3*+/− group showed a more irregular pattern of ventricular oscillations (Figure 7A). Furthermore, although at larval stages, the heart rates of the mutants were comparable to the control group. In contrast, in adults, interestingly, the heterozygous *mybpc3*+/− adult zebrafish group showed a significantly reduced heart rate (Figure 7B). The observed irregular pattern of the ventricular oscillations in the adult *mybpc3*+/− group was also associated with a significantly reduced heart rate at 61.5 bpm, *p* < 0.0001, when compared to both the *mybpc3*−/− and control groups’ heart rates at 88.5 bpm and 87.6 bpm, respectively.

### 2.5. Reduced Adult Swimming Performance in mybpc3 Mutant Zebrafish

The adult *mybpc3*+/− zebrafish model was examined for its cardiac function. The reduced heart rates of the adult mutant zebrafish resulted in a decreased ability to swim in the endurance experiment. Both the calculated cumulative duration of moving speed to nonmoving for the heterozygous *mybpc3*+/− group were significantly lower and reduced compared to the homozygous *mybpc3*−/− mutant fish control group. The movement ability of both the *mybpc3*−/− and *mybpc3*+/− adult fish was associated with reduced swimming distance and velocity (Figure 7C–E). The total distance and velocity decreased in the *mybpc3* mutant fish; however, the heterozygous *mybpc3*+/− group showed a more significant decrease when compared to the control fish (Figure 7D,E).

### 2.6. Differentially Expressed Genes through the Development of Hypertrophic Cardiomyopathy

**Figure 8:**
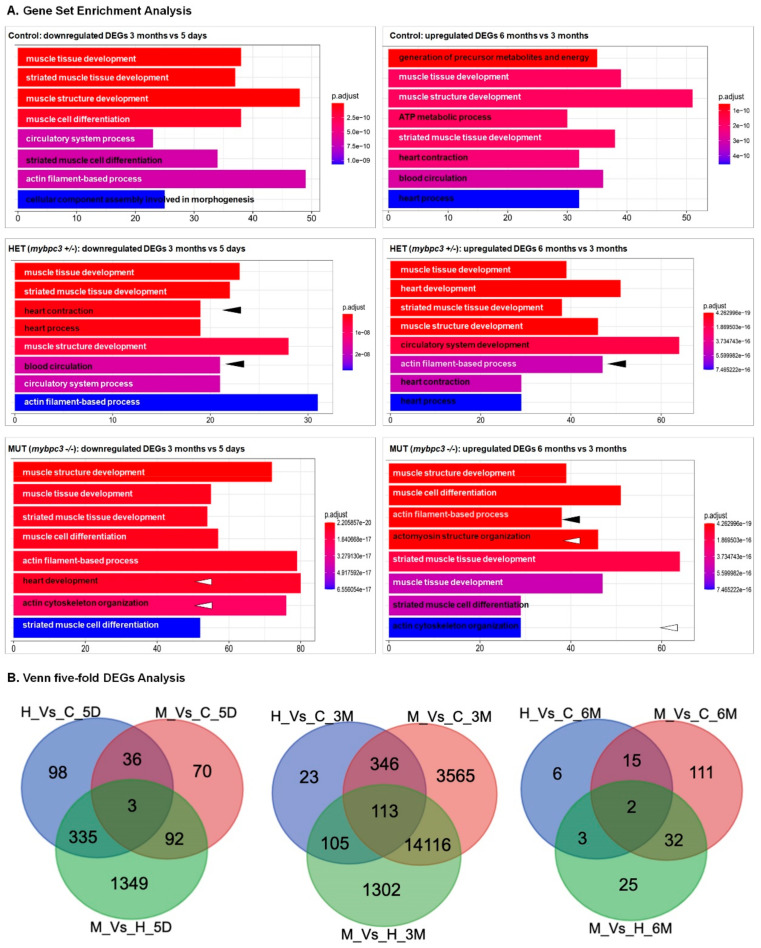
A Venn diagram illustrating the differentially expressed genes (DEGs). (**A**) DEGs were examined for the groups across heart development (time: 5 days, 3 months, and 6 months). (**B**) DEGs whose expression was changed ≥1-fold throughout normal physiologic growth (control (wild-type), red circle), or hypertrophic growth (*mypbc3*+/−, blue circle; *mypbc3*−/−, green circle). Subsections of the Venn diagram include genes that are exclusively regulated during physiologic growth, regulated during both physiologic and hypertrophic growth, and regulated exclusively during hypertrophic growth. The values listed indicate the number of genes that belong to each section. The genes that comprise each subsection of the Venn diagram are available in Supplementary Table S1 (cardiac physiologic growth), and Supplementary Table S2 (cardiac hypertrophic growth).

To investigate the cell signaling mechanisms that may influence the differential pathways leading to physiologic growth vs. pathologic hypertrophy and to highlight differential RNA expression before and after the presentation of overt hypertrophy and dysfunction, we performed RNA-Seq analysis using the total RNA isolated from the hearts of *mybpc3*−/−, *mybpc3*+/−, and the control (wild-type) zebrafish groups at different developmental stages, comparing the heart average physiologic vs. hypertrophic growth. Development of the cardiac conduction system in zebrafish [42] processes were selected as the study time points; the examination of five-day-old larvae at the cardiac looping trabeculation versus adult zebrafish at three-months old and at six-months old to follow HCM disease progression through life. We did find gene expression differences between the examined groups. Additionally, Gene Ontology enrichment and the KEGG analysis of mRNAs were conducted to identify the biological modules and pathological pathways associated with the differentially expressed genes (DEGs). Through heart development from larvae stage (5-days old) to adult stage (3-months old), we did find gene expression differences between the examined groups (Figure 8A). All groups shared the downregulation of the following biological processes: muscle structure and tissue development, striated muscle development, and actin filament-based process (Figure 8B). However, the heterozygous *mybpc3*+/− group differed in the downregulation of the heart process and contraction biological processes, the circulatory system process, and blood circulations. In contrast, the homozygous *mybpc3*−/− group differed in the downregulation of heart development and in the biological processes of actin cytoskeleton organization. Interestingly, in the early stages of cardiac development in 5-day old larvae, the *mybpc3*−/− versus *mybpc3*+/− showed specific enrichment of 1349 DEGs involved in protein translation, protein catabolic process, and peptide biosynthetic process (Supplementary Figure S2). Following disease progression from 3 to 6 months, all groups shared the upregulation of physiological and biological processes: muscle structure and tissue development, striated muscle development, and heart development (Figure 8A). The HCM zebrafish model deviated in the transcriptome profile through hypertrophic heart growth. The heterozygous *mybpc3*+/− group differed in the upregulation of the biological process of the circulatory system development. Both the homozygous and heterozygous, *mybpc3*−/− and *mybpc3*+/−, groups differed in the upregulation of the heart process and contraction and cardiac muscle tissue development biological processes. Remarkably, at later stages, the zebrafish *mybpc3*−/− model at 3-months of age showed 23 DEGs that matched 14 hypertrophy-associated genes and other genes from the calcium-handling, and the NMD prespecified pathway defined associated genes (igf1ra, itpr1a, jak1, kras, mapk1, mef2aa, mtor, nf1b, nfatc3a, ptena, raf1a, rnps1, slc8a1b, stim1b, tgfbr2a, trpm4b.2, upf3b, ednrab, erbb2, fgfr1bl, and atf2). These results demonstrate that these genes primarily regulate cardiovascular system development and cardiac physiology through the *mybpc3*-related HCM.

## 3. Discussion

HCM *c-MYBPC3*-related early-onset cardiac impairment associated with poor prognosis is a growing health care burden [43]. We employed the zebrafish model organism to studying cardiac remodeling in the early stages of HCM heart disease by establishing the first zebrafish stable *mybpc3*-HCM model. In this study, the mutant pathogenesis of cardiac remodeling in zebrafish larvae recapitulated perspectives of hypertrophic cardiomyopathy in mammals as *mybpc3* KO contributed to early adverse cardiac events. The results presented here indicate that *mybpc3* is essential for early cardiac development and cardiac function in zebrafish larvae. These mutants displayed restrictive compact cardiac chambers at the initial stages of development. The model demonstrated that cardiomyocyte hyperplasia is one of the early stages of cardiac remodeling, thus, highlighting the effects of cardiomyocyte hyperplasia on cardiac remodeling and its contribution to HCM to be studied. In the current study, at the larval stages, the *mypbc3*-KO zebrafish displayed significant morphological heart alterations related to a significant decrease in the ventricular and atrial diameters at both systolic and diastolic states (Figure 3 and Figure 4). Remarkably, when we examined the growth of the heart chambers, the mutant myocardial wall was thicker and more compact in comparison to the control. Similarly, cardiomyocyte hypertrophy or hyperplasia was shown to contribute to the cardiac remodeling process in different mammalian models [44,45,46]. An earlier study utilizing the microarray analysis of the HCM MyBP-C−/− mice model revealed an increase in the cell proliferation markers that were associated with early cardiac hyperplasia and contributed to hypertrophic cardiac growth [47]. Our study showed that the KO of *mybpc3* can alter cardiomyocyte biology at the very early stages of heart development. The increased number of cardiomyocytes and the restrictive cardiac chamber diameters detected in the zebrafish *mybpc3* KO imply that myocyte hyperplasia is a physiological effect that is associated with the chronic ventricular myocardial wall stress in zebrafish hearts. Consistently, previous studies have shown that heterozygous mutant mice expressing a truncated form of *Mybpc (*Mybpc3^t/+^) have a normal morphology, but are more prone to developing greater left ventricular hypertrophy than wild-type mice [48,49].

Functionally, although the zebrafish *mybpc3* KO cardiac contractility was similar to the wild-type control, the ejection fraction was significantly increased in the *mypbc3* mutants (Figure 4). At the larval stage, the zebrafish mutants demonstrated an early cardiac phenotype of myocardium remodeling, evident by concurrent cardiomyocyte hyperplasia and increased ejection fraction, suggesting these critical processes in HCM initiation to counteract the increased ventricular myocardial wall stress (Figure 4). Consistently, homozygous mutant mice expressing a truncated form of *Mybpc (*Mybpc3^t/t^) have significantly increased left ventricular volumes and hypertrophy (increased left ventricular wall thickness) due in part to increased numbers of cardiomyocytes (hyperplasia) from additional perinatal cell divisions [50]. It was presented earlier that transcriptome analysis of cardiac hypertrophic *Mybpc3*-null mice demonstrated contractile changes related to the absence of MyBP-C at the heart sarcomere level [51]. Likewise, earlier studies have revealed that the mechanical stress induced by cardiac pressure overload may lead to early ventricular hypertrophic remodeling during cardiac stress [51,52]. It has been shown that the deletion of *Mybpc3* increases ventricular shortening velocity, force output, and force redevelopment [53,54,55]. The impact of *Mybpc3* deletion on the left atrium was more prolonged Ca2+ transient and sarcomere shortening in the *Mybpc3* knockout mice [56]. As a result of the enhanced myofilament Ca2+ sensitivity, a deficit in diastolic relaxation and a reduced dynamic range of cell shortening were observed [56,57]. Later through cardiac development, our assessment of the genetic *mybpc3* knockout impact on the larvae cardiac development and function culminated this early cardiac phenotype into specific hypertrophic cardiomyopathy seen in adult zebrafish model. The histological examination of adult zebrafish revealed hypertrophic ventricular growth and one impairment is the cardiac function in both heterozygous and homozygous groups. Additionally, the heterozygous group average heart rate was significantly decreased, and interestingly, the heterozygous group showed a more significant reduction in swimming total distance and velocity and cumulative movements after exercise.

It is established that MyBP-C is a crucial regulator of actin-myosin contractile dynamics [58]. The contraction and relaxation of cardiac muscle are mediated by the sliding of interdigitating thick filaments (myosin) and thin filaments (actin). In addition, the sarcomere also contains several accessory proteins that are involved in the assembly, maintenance of structural integrity, and regulation of contractile activity of the heart. Our transcriptome analysis using dissected zebrafish hearts revealed that actin is the critical component that is directly associated with the molecular mechanism involved in the pathogenesis of *mybpc3*-related HCM (Figure 6, Figure 7 and Figure 8). Our transcriptome data of 5-day old larvae of *mybpc3*−/− zebrafish model supported the in vitro model of the *MYBPC3*-mutant cell lines of patient-engineered iPSCMs in which the protein folding-associated gene pathways were significantly dysregulated (Supplementary Figure S2) {Helms, 2020 #64}. While at 3-months of age, the *mybpc3*−/− zebrafish model demonstrated calcium-handling, NMD, and hypertrophic gene dysregulation associated with HCM.

Distinctively, the zebrafish *mybpc3-*induced cardiac hypertrophic mutants uniquely dysregulated pathways associated with the actin filament-based process of actin cytoskeleton organization through cardiac development and HCM progression in the zebrafish model.

Overall, our present molecular and cellular analysis of cardiac development/function has further improved our understanding of pathogenic signaling pathways in inherited hypertrophic cardiomyopathies. Our *mybpc3* zebrafish model exhibited an early cardiac phenotype of myocardium remodeling, associated with concurrent cardiomyocyte hyperplasia and increased ejection fraction, suggesting that *mybpc3* KO-induced hyperplasia may be a key mechanism in HCM initiation. The alterations in gene expression patterns also highlighted the downregulation of the actin cytoskeleton organization followed by the upregulation of heart contraction through HCM progression. These processes can be further investigated for biomarkers of HCM progression or can be targeted for potential therapeutics. Furthermore, our established zebrafish model can serve as a basic genetic background model to further study the biological functions of *mybpc3* in an in vivo system or to study specific alleles of human *c-MYBPC3* variants observed in patients to unravel the molecular mechanisms that lead to cardiac dysfunction.

## 4. Materials and Methods

### 4.1. Zebrafish Lines and Maintenance

Zebrafish (*Danio rerio*) were raised and maintained, at the Sidra Medicine, Research Department, Doha, Qatar, following the procedures described in the zebrafish book [59]. Adult zebrafish were kept within a circulating system (Pentair Aquatic Habitats, Apopka, FL, USA) on cycles of 14 h light, 10 h dark at 28 °C, and fed Artemia nauplii (Invy, Cat # BS-90, Aquaculture, USA). Wild-type zebrafish embryos were obtained according to the scheduled breeding and staged [60]. All experiments were carried out according to the protocols approved by Qatar University IACUC Office regulations (QU-IACUC 2-9/2019-1).

### 4.2. TALEN and Targeting-Vector Construction for mybpc3

A CLC sequence viewer was used to obtain the alignments and phylogenetic tree for cardiac myosin-binding protein sequences from different species. The cardiac MyBP-C protein sequences from the following species were used for alignment: human (*Homo sapiens*, NM_000256, NP_000247), rat (*Rattus norvegicus*, NM_001106490, NP_001099960), mouse (*Mus musculus*, NM_008653, NP_032679), cat (*Felis catus*, M3VYP3), chicken (*Gallus*, NM_205116, NP_990447), zebrafish (*Danio rerio*, NM_001044349, NP_001037814), and frog (*Xenopus tropicalis*, F6VSF7).

To generate a *mybpc3* zebrafish knock-out, TALEN targeting *mybpc3* (ENSDARG00000011615) located at chromosome 7, exon 2, was selected as the targeted site to introduce a premature stop-codon. The target gene *mybpc3* (ENSDARG00000011615) sequence is a follows:

‘ATGCCAGAGCCAACTAAAAAAATAGTCTCAGCTTTCAGCAAGAAACCAAAGTCCCAGACTGCTGAGATTGGGGCAAGAGTCATATTTGAAGCTGAAACTGAGAAGCCAGGTGTAAAAGTGAAATGGCAGCGAGAATCCAAGGACATCTTACCCAGTCACAAATACACCATCTCAGCAGAGGACAATAAGCATTCTCTCACTATAAATAAT’

The left (L) recognition sequence (16 bp): GTAAAAGTGAAATGGC; the right (R) recognition sequence (18 bp): GTGACTGGGTAAGATGTC. A total of 10 ng/µL of TALEN RNAs were injected into the cytoplasm of one-cell stage zebrafish embryos.

### 4.3. TALEN Efficiency in Zebrafish Embryos

To test the *mybpc3* TALEN vector efficiency, the genomic DNA was isolated from 30–50 injected zebrafish embryos. Fragments containing the TALEN target site were amplified by PCR using genomic DNA-specific primers. Then, the PCR product was digested with a specific BsaI-HF endonuclease (New England BioLabs, Cat # R3733S, Ipswich, MA, USA) restriction enzyme to examine the efficiency of TALEN.

### 4.4. Genotyping of TALEN Edited mybpc3 Zebrafish Line

The adult tail fin or tail clips of zebrafish were incubated in 100 μL extraction buffer containing 50 mM Tris-HCl (pH 8.5) (Roche, Cat # 1081284601, Mannheim, Germany), 1 mM EDTA (Invitrogen, Cat # 15575-020, Thermo Fisher Scientific, Waltham, MA, USA), 0.5% Tween-20 (Sigma, Cat # P2287, St. Louis, MO, USA), and 80 mg/mL proteinase K (Ambion, Life Technologies, Austin, TX, USA) for 3–4 h at 55 °C to extract its genomic DNA. Proteinase K (Invitrogen, Cat # 46-6084, Thermo Fisher Scientific, Waltham, MA, USA) was inactivated by heating to 95 °C for 10 min. Then, 1 μL of the genomic DNA mix was used in a standard 50 μL PCR reaction with Phusion High-Fidelity DNA polymerase (New England BioLabs, Cat # M0530S, Ipswich, MA, USA) and primers spanning the TALEN target site (Fw: 5′AAATGTCATATATAAATGATCTTG3′; Rv: 5′CATAAGGACGTAGATGAAGTGGAA3′) according to the manufacturer’s instructions. PCR products were purified using the Qiagen QIAquick PCR Purification Kit (Qiagen, Cat #: 28104, Germantown, MD, USA) and digested using BsaI-HF endonuclease (New England BioLabs, Ipswich, MA, USA) overnight at 37 °C in a 10 μL reaction, according to the manufacturer’s instructions. Samples were then run on a 2% agarose gel. Mutant products were undigested at 450 bp, whereas digested wild-type products were detected as fragments of 225 bp. As per the manufacturer’s instructions, the PCR products were purified using QIAquick purification columns (Qiagen, Cat # 28115, Germantown, MD, USA), sequenced, and aligned to the wild-type genomic sequence. Founder zebrafish containing deletions were identified by genotyping with restriction enzymes, followed by sequencing to determine the precise molecular nature of the genomic change. Founder zebrafish with the *mybpc3* mutation were outcrossed to wild-type zebrafish to produce an F1 generation, which was then used for breeding to generate homozygous mutants. Homozygous mutant fish were identified by PCR, restriction fragment, and genotyping.

### 4.5. Generation of Zebrafish mybpc3 Mutant with Green-Fluorescent Hearts

Injected F0 embryos were raised to adulthood and outcrossed with wild-type zebrafish to produce F1. The F1 embryos were raised to adulthood, then genotyped, homozygous KO *mybpc3* zebrafish were outcrossed to Transgenic Tg (*cmlc2*:eGFP), which expresses a heart-specific green fluorescent protein (GFP). Embryos were raised to adulthood and in-crossed to produce *mybpc3*+/−::*cmlc2*:eGFP, which were raised to be in-crossed to create the F2 generation of *mybpc3*−/−::*cmlc2*:eGFP that exhibited an intense expression of heart GFP to adulthood.

### 4.6. Microscopic Assessment of Cardiac Phenotypes

At 72 hpf, the heartbeats of different zebrafish groups were recorded at high resolution using Stereomicroscope Zeiss LUMAR.V12 (Zeiss, Jena, Germany) at 60 frames per second (fps). Haloperidol (Sigma, Cat # H1512-5G, St. Louis, MO, USA), a first-generation antipsychotic known to be cardiotoxic in zebrafish and humans, was used as a positive control [61,62,63]. Cardiac heart chamber measurements were assessed using Danioscope software (Noldus Technologies, Wageningen, The Netherlands). To minimize the effect of environmental temperature on cardiac function, zebrafish larvae were kept in individual wells and removed from the 28 °C incubator immediately before cardiac measurement. In addition, zebrafish larvae were mounted in 3% methylcellulose (Sigma, Cat # M0387, St. Louis, MO, USA) before imaging for stabilization.

### 4.7. Assessment of Cardiovascular Performance

The cardiac contractility of the zebrafish hearts was assessed using Danioscope software (Noldus Technologies, Wageningen, The Netherlands). For the heart rate and rhythm analysis of the embryonic heart, videos were analyzed of beating ventricles and atria for 72 hpf embryos at 60 fps.

### 4.8. Immunostaining of Zebrafish Mutants and Cardiomyocytes Count

Whole-mount immunofluorescence was performed as previously described [64,65]. Briefly, *mybpc3*−/−::*cmlc2*:eGFP zebrafish expressing GFP in their cardiomyocytes under the control of the cmlc2 promotor at 72 hpf were fixed in 4% paraformaldehyde (Sigma, Cat # P6148, St. Louis, MO, USA) and then washed with 1X PBS (Sigma, Cat #P-5493, St. Louis, MO, USA) containing 0.1% Tween (PBST). Larvae were incubated in a blocking solution (PBST supplemented with 2% bovine serum albumin (BSA) (VWR, Cat # 0332, Solon, OH, USA) and 10% goat serum (Sigma, Cat # G9023, St. Louis, MO, USA)) for 1.5 h at room temperature (RT). Then, the surface marker for ventricular cardiomyocytes, mouse anti-ZN-8 (Hybridoma bank; 1:50 (Developmental Studies Hybridoma Bank, Houston, TX, USA)), was added to the zebrafish larvae overnight at 4 °C in a blocking solution. Afterward, the anti-mouse Alexa594 (Invitrogen; 1:400, Cat # A32744, Thermo Fisher Scientific, Waltham, MA, USA) was added as a secondary antibody and incubated with DAPI (1:1000) for 5 min. Larvae were imaged using an SP8 Leica confocal microscope for cardiomyocyte quantification.

### 4.9. The Heart Rate of Adult Zebrafish

Anesthetic preparation: MS-222 (Tricaine methanesulfonate) is a widely used anesthetic material in zebrafish. As previously reported, MS-222 affects skeletal muscle and cardiac muscle function, which is cardiotoxic and reduces the heart rate [66]. Therefore, a combination of MS-222 (Western Chemical Inc., Cat #: NC0872873, Ferndale, WA, USA) and isoflurane (Baxter, Cat #: FDG9621ME, Deerfield, IL, USA) as an anesthetic solution was prepared via a modified protocol [67] for zebrafish with minimal cardiac consequence function effects. The solution was prepared with a combination of 0.1 mL of a buffered MS-222 stock solution “4 mg/mL” and 1.5 mL of isoflurane dissolved in 100 mL of fish water. Individual fish were gently poured inside the solution until the zebrafish had no response. Then, the heart rate was directly imaged for 10 s using a ZEISS microscope (Model Stemi 2000-C) with a IMAGINGSOURCE camera (The ImagingSource, Model 33UX252, Bremen, Germany). The recorded videos of each examined zebrafish larvae were used to calculate the individual heart rate and cardiac activity percentage by DanioScope software (video-based analysis tool).

### 4.10. Assessment of Swimming Performance and Endurance of Adult Zebrafish

The zebrafish swimming behavior was assessed using video-based tracking software that measures the swimming parameters for swimming analysis such as velocity, acceleration, and distance moved during the trial. Swimming behavioral tests were performed as previously described with some modifications on adult zebrafish. The fish was placed in a 3.5 L zebrafish housing tank from Techniplast and allowed to acclimatize for 5 min inside the system. Then, the water speed flow was increased to the maximum of 55 mL\s for 30 min. After that, the fish was gently poured into the observation tank for live tracking records using the EthoVision XT7 system (version 13, Noldus, Wageningen, The Netherlands). Two arenas were defined as the top and side view; acquisition started after subject detection within the arena as defined in the trial control settings. The fish was tracked at a 30 fps rate for 1 min immediately after the fish was poured into the observation tank.

### 4.11. Identification of Differentially Expressed Genes (DEGs) between HCM Zebrafish Mutants and Healthy Controls

Dissected zebrafish hearts were collected at different time points of development: 5 days, 3 months, and 6 months. The total RNA was extracted from pooled hearts for five days (32 hearts), 3 months (24 hearts), and 6 months (12 hearts) using the Qiagen RNeasy Plus Micro Kit (QIAGEN, Cat #: 74034, Germantown, MD, USA). Three replicates were submitted for RNA sequencing for each examined group of the control, mutant, and heterozygous.

The mRNA libraries were prepared from the total RNA using the QuantSeq 3’mRNA-Seq Library Prep Kit-FWD (Lexogen, Cat #: 015.96, Cambridge Bioscience, Cambridge, UK) according to the manufacturer’s instructions. The first strand was synthesized using Oligo-dT primers, followed by RNA removal and the synthesis of complementary strand (second strand synthesis) initiated by random priming. Afterward, the Illumina- or IonTorrent-specific linker sequences were introduced by primers. The resulting double-stranded cDNA was purified with magnetic beads included in the kit. Then, the libraries were amplified for 18 PCR cycles and labeled with different single indices. The quality and size of the libraries were determined using the NGS 3K assay on the Labchip GXII (Perkin Elmer, Waltham, MA, USA) and pooled based on quantification via qPCR using the KAPA HiFi Library Quantification Kit on a Roche LightCycler 480 (Roche Sequencing and Life Science, KK8401 Cat #: 07962169001, Kapa Biosystems, Indianapolis, IN, USA). The final library pool was sequenced on an Illumina NextSeq 500 with a High Output 75 bp Single End Read Kit, at a depth of 8 million reads per library.

The RNA sequencing data in the fastq format was first mapped to the zebrafish (*Danio rerio*) genome assembly GRCz11 (danRer11) (https://www.ncbi.nlm.nih.gov/grc/zebrafish, accessed on 17 September 2020) using the STAR aligner (v2.6.1d) (https://github.com/alexdobin/STAR, accessed on 17 September 2020). Read summarization was performed using feature counts (v2.0.0) (http://subread.sourceforge.net/, accessed on 17 September 2020). Differential expression analysis was performed using the limma package (https://bioconductor.org/packages/release/bioc/html/limma.html, accessed on 17 September 2020). Gene Set Enrichment Analysis was performed using the Cluster Profiler package (https://bioconductor.org/packages/release/bioc/html/clusterProfiler.html, accessed on 17 September 2020). To assess the DEGs, the gene expression differences between the HCM and normal controls were compared. The list of genes was identified as DEG protein-coding genes based on the cut-off of the false discovery rate (FDR) < 0.05 (Figure 8).

### 4.12. Statistical Analysis

The values displayed in each graph are the mean ± standard deviation. Statistical analysis was performed using GraphPad Prism 7. One-way ANOVA was used to analyze multiple group comparisons. The Student *t*-test was used for differences between the two groups. Significant differences between groups were expressed using *p* values: * *p* < 0.05, ** *p* < 0.01, *** *p* < 0.001, **** *p* < 0.0001.

## Data Availability

The data presented in this study are available on request from the corresponding author.

## Supplementary Materials

The following supporting information can be downloaded at: https://www.mdpi.com/article/10.3390/ijms23168840/s1.

## References

[1] Semsarian C., Ingles J., Maron M.S., Maron B.J. (2015). New perspectives on the prevalence of hypertrophic cardiomyopathy. J. Am. Coll. Cardiol..

[2] Maron B.J., Gardin J.M., Flack J.M., Gidding S.S., Kurosaki T.T., Bild D.E. (1995). Prevalence of hypertrophic cardiomyopathy in a general population of young adults. Echocardiographic analysis of 4111 subjects in the CARDIA study. Coronary artery risk development in (young) adults. Circulation.

[3] Basit H., Brito D., Sharma S. (2022). Hypertrophic Cardiomyopathy. https://www.ncbi.nlm.nih.gov/books/NBK430788/.

[4] Nagueh S.F., Bachinski L.L., Meyer D., Hill R., Zoghbi W.A., Tam J.W., Quiñones M.A., Roberts R., Marian A. (2001). Tissue doppler imaging consistently detects myocardial abnormalities in patients with hypertrophic cardiomyopathy and provides a novel means for an early diagnosis before and independently of hypertrophy. Circulation.

[5] Maron B.J., Ommen S.R., Semsarian C., Spirito P., Olivotto I., Maron M.S. (2014). Hypertrophic cardiomyopathy: Present and future, with translation into contemporary cardiovascular medicine. J. Am. Coll. Cardiol..

[6] Marian A.J., Braunwald E. (2017). Hypertrophic cardiomyopathy: Genetics, pathogenesis, clinical manifestations, diagnosis, and therapy. Circ. Res..

[7] Schlossarek S., Mearini G., Carrier L. (2011). Cardiac myosin-binding protein C in hypertrophic cardiomyopathy: Mechanisms and therapeutic opportunities. J. Mol. Cell. Cardiol..

[8] Antunes M.d.O., Scudeler T.L. (2020). Hypertrophic cardiomyopathy. Int. J. Cardiol. Heart Vasc..

[9] Sen-Chowdhry S., Jacoby D., Moon J., McKenna W.J. (2016). Update on hypertrophic cardiomyopathy and a guide to the guidelines. Nat. Rev. Cardiol..

[10] Sedaghat-Hamedani F., Kayvanpour E., Tugrul O.F., Lai A., Amr A., Haas J., Proctor T., Ehlermann P., Jensen K., Katus H.A. (2018). Clinical outcomes associated with sarcomere mutations in hypertrophic cardiomyopathy: A meta-analysis on 7675 individuals. Clin. Res. Cardiol..

[11] Tsang D.C., Link M.S. (2021). Sudden cardiac death in athletes. Tex. Heart Inst. J..

[12] Mohamed I.A., Krishnamoorthy N., Nasrallah G.K., Da’As S.I. (2017). The role of cardiac myosin binding protein C3 in hypertrophic cardiomyopathy-progress and novel therapeutic opportunities. J. Cell. Physiol..

[13] Maron B.J. (2018). Clinical course and management of hypertrophic cardiomyopathy. N. Engl. J. Med..

[14] Maron B.J., Maron M.S., Semsarian C. (2012). Genetics of hypertrophic cardiomyopathy after 20 years: Clinical perspectives. J. Am. Coll. Cardiol..

[15] Vikhorev P.G., Vikhoreva N.N. (2018). Cardiomyopathies and related changes in contractility of human heart muscle. Int. J. Mol. Sci..

[16] Walsh R., Buchan R., Wilk A., John S., Felkin L.E., Thomson K., Chiaw T.H., Loong C.C.W., Pua C.J., Raphael C. (2017). Defining the genetic architecture of hypertrophic cardiomyopathy: Re-evaluating the role of non-sarcomeric genes. Eur. Heart J..

[17] Walsh R., Thomson K.L., Ware J.S., Funke B.H., Woodley J., McGuire K.J., Mazzarotto F., Blair E., Seller A., Taylor J.C. (2017). Reassessment of Mendelian gene pathogenicity using 7855 cardiomyopathy cases and 60,706 reference samples. Genet. Med..

[18] Harper A.R., Goel A., Grace C., Thomson K.L., Petersen S.E., Xu X., Waring A., Ormondroyd E., Kramer C.M., HCMR Investigators (2021). Common genetic variants and modifiable risk factors underpin hypertrophic cardiomyopathy susceptibility and expressivity. Nat. Genet..

[19] Kassem H.S., Azer R.S., Ayad M.S., Moharem-Elgamal S., Magdy G., Elguindy A., Cecchi F., Olivotto I., Yacoub M.H. (2012). Early results of sarcomeric gene screening from the egyptian national BA-HCM program. J. Cardiovasc. Transl. Res..

[20] Heling L.W.H.J., Geeves M.A., Kad N.M. (2020). MyBP-C: One protein to govern them all. J. Muscle Res. Cell Motil..

[21] Hou L., Kumar M., Anand P., Chen Y., El-Bizri N., Pickens C.J., Seganish W.M., Sadayappan S., Swaminath G. (2022). Modulation of myosin by cardiac myosin binding protein-C peptides improves cardiac contractility in ex-vivo experimental heart failure models. Sci. Rep..

[22] Luther P.K., Vydyanath A. (2011). Myosin binding protein-C: An essential protein in skeletal and cardiac muscle. J. Muscle Res. Cell Motil..

[23] Finley N.L., Cuperman T.I. (2014). Cardiac myosin binding protein-C: A structurally dynamic regulator of myocardial contractility. Pflüg. Arch. Eur. J. Physiol..

[24] Pfuhl M., Gautel M. (2012). Structure, interactions and function of the N-terminus of cardiac myosin binding protein C (MyBP-C): Who does what, with what, and to whom?. J. Muscle Res. Cell Motil..

[25] Al-Khayat H.A. (2013). Three-dimensional structure of the human myosin thick filament: Clinical implications. Glob. Cardiol. Sci. Pract..

[26] Lin B.L., Li A., Mun J.Y., Previs M.J., Previs S.B., Campbell S.G., Dos Remedios C.G., Tombe P.D.P., Craig R., Warshaw D.M. (2018). Skeletal myosin binding protein-C isoforms regulate thin filament activity in a Ca^2+^-dependent manner. Sci. Rep..

[27] Shaffer J.F., Kensler R.W., Harris S.P. (2009). The myosin-binding protein C motif binds to F-actin in a phosphorylation-sensitive manner. J. Biol. Chem..

[28] Belknap B., Harris S.P., White H.D. (2014). Modulation of thin filament activation of myosin ATP hydrolysis by N-terminal domains of cardiac myosin binding protein-C. Biochemistry.

[29] Suay-Corredera C., Pricolo M.R., Velázquez-Carreras D., Pathak D., Nandwani N., Pimenta-Lopes C., Sánchez-Ortiz D., Urrutia-Irazabal I., Vilches S., Dominguez F. (2021). Nanomechanical phenotypes in cardiac myosin-binding protein C mutants that cause hypertrophic cardiomyopathy. ACS Nano.

[30] Kampourakis T., Yan Z., Gautel M., Sun Y.-B., Irving M. (2014). Myosin binding protein-C activates thin filaments and inhibits thick filaments in heart muscle cells. Proc. Natl. Acad. Sci. USA.

[31] Coulton A.T., Stelzer J.E. (2012). Cardiac myosin binding protein C and its phosphorylation regulate multiple steps in the cross-bridge cycle of muscle contraction. Biochemistry.

[32] Sadayappan S., Gulick J., Osinska H., Barefield D., Cuello F., Avkiran M., Lasko V.M., Lorenz J.N., Maillet M., Martin J.L. (2011). A critical function for Ser-282 in cardiac myosin binding protein-C phosphorylation and cardiac function novelty and significance. Circ. Res..

[33] Seeger T., Shrestha R., Lam C.K., Chen C., McKeithan W.L., Lau E., Wnorowski A., McMullen G., Greenhaw M., Lee J. (2018). A premature termination codon mutation in MYBPC3 causes hypertrophic cardiomyopathy via chronic activation of nonsense-mediated decay. Circulation.

[34] Carrier L., Mearini G., Stathopoulou K., Cuello F. (2015). Cardiac myosin-binding protein C (MYBPC3) in cardiac pathophysiology. Gene.

[35] Wijnker P.J.M., van der Velden J. (2020). Mutation-specific pathology and treatment of hypertrophic cardiomyopathy in patients, mouse models and human engineered heart tissue. Biochim. Biophys. Acta BBA Mol. Basis Dis..

[36] Lu Y., Kwan A., Jeffries C.M., Guss M., Trewhella J. (2012). The motif of human cardiac myosin-binding protein C is required for its Ca^2+^-dependent interaction with calmodulin. J. Biol. Chem..

[37] Mun J.Y., Gulick J., Robbins J., Woodhead J., Lehman W., Craig R. (2011). Electron microscopy and 3D reconstruction of F-actin decorated with cardiac myosin-binding protein C (cMyBP-C). J. Mol. Biol..

[38] Poon K.L., Brand T. (2013). The zebrafish model system in cardiovascular research: A tiny fish with mighty prospects. Glob. Cardiol. Sci. Pract..

[39] Amsterdam A., Hopkins N. (2006). Mutagenesis strategies in zebrafish for identifying genes involved in development and disease. Trends Genet..

[40] Da’as S.I., Yu J., Butcher J.T., Krishnamoorthy N., Al Suwaidi J.A.S., Kassem H., Al Shafai K.N., Al-Hashemi M.A., Shuayb L., Brand T. (2014). Different human mutations in the myosin binding protein C3 (MYBPC3) produce specific cardiac phenotypes in the zebrafish. Circulation.

[41] Liu X., Jiang T., Piao C., Li X., Guo J., Zheng S., Zhang X., Cai T., Du J. (2015). Screening mutations of MYBPC3 in 114 unrelated patients with hypertrophic cardiomyopathy by targeted capture and next-generation sequencing. Sci. Rep..

[42] Chi N.C., Shaw R.M., Jungblut B., Huisken J., Ferrer T., Arnaout R., Scott I., Beis D., Xiao T., Baier H. (2008). Genetic and physiologic dissection of the vertebrate cardiac conduction system. PLoS Biol..

[43] Sabater-Molina M., Pérez-Sánchez I., Hernandez Del Rincon J.P., Gimeno J.R. (2018). Genetics of hypertrophic cardiomyopathy: A review of current state. Clin. Genet..

[44] Frey N., Luedde M., Katus H.A. (2012). Mechanisms of disease: Hypertrophic cardiomyopathy. Nat. Rev. Cardiol..

[45] Von Gise A., Lin Z., Schlegelmilch K., Honor L.B., Pan G.M., Buck J.N., Ma Q., Ishiwata T., Zhou B., Camargo F.D. (2012). YAP1, the nuclear target of Hippo signaling, stimulates heart growth through cardiomyocyte proliferation but not hypertrophy. Proc. Natl. Acad. Sci. USA.

[46] Bravo P.E., Pinheiro A., Higuchi T., Rischpler C., Merrill J., Santaularia-Tomas M., Abraham M.R., Wahl R.L., Abraham T.P., Bengel F.M. (2012). PET/CT Assessment of symptomatic individuals with obstructive and nonobstructive hypertrophic cardiomyopathy. J. Nucl. Med..

[47] Farrell E.T., Grimes A.C., De Lange W.J., Armstrong A.E., Ralphe J.C. (2017). Increased postnatal cardiac hyperplasia precedes cardiomyocyte hypertrophy in a model of hypertrophic cardiomyopathy. Front. Physiol..

[48] McConnell B.K., Jones K.A., Fatkin D., Arroyo L.H., Lee R.T., Aristizabal O., Turnbull D.H., Georgakopoulos D., Kass D., Bond M. (1999). Dilated cardiomyopathy in homozygous myosin-binding protein-C mutant mice. J. Clin. Investig..

[49] Barefield D., Kumar M., Gorham J., Seidman J.G., Seidman C.E., de Tombe P.P., Sadayappan S. (2015). Haploinsufficiency of MYBPC3 exacerbates the development of hypertrophic cardiomyopathy in heterozygous mice. J. Mol. Cell. Cardiol..

[50] Jiang J., Burgon P.G., Wakimoto H., Onoue K., Gorham J.M., O’Meara C.C., Fomovsky G., McConnell B.K., Lee R.T., Seidman J.G. (2015). Cardiac myosin binding protein C regulates postnatal myocyte cytokinesis. Proc. Natl. Acad. Sci. USA.

[51] Farrell E., Armstrong A.E., Grimes A.C., Naya F.J., De Lange W.J., Ralphe J.C. (2018). Transcriptome analysis of cardiac hypertrophic growth in MYBPC3-null mice suggests early responders in hypertrophic remodeling. Front. Physiol..

[52] Lyon R.C., Zanella F., Omens J.H., Sheikh F. (2015). Mechanotransduction in cardiac hypertrophy and failure. Circ. Res..

[53] Korte F.S., McDonald K.S., Harris S.P., Moss R.L. (2003). Loaded shortening, power output, and rate of force redevelopment are increased with knockout of cardiac myosin binding protein-C. Circ. Res..

[54] Kulikovskaya I., McClellan G., Flavigny J., Carrier L., Winegrad S. (2003). Effect of MyBP-C binding to actin on contractility in heart muscle. J. Gen. Physiol..

[55] Stelzer J.E., Dunning S.B., Moss R.L. (2006). Ablation of cardiac myosin-binding protein-C accelerates stretch activation in murine skinned myocardium. Circ. Res..

[56] Pohlmann L., Kroger I., Vignier N., Schlossarek S., Krämer E., Coirault C., Sultan K.R., El-Armouche A., Winegard S., Eschenhagen T. (2007). Cardiac myosin-binding protein C is required for complete relaxation in intact myocytes. Circ. Res..

[57] Van Dijk S.J., Paalberends E.R., Najafi A., Michels M., Sadayappan S., Carrier L., Boontje N.M., Kuster D.W., van Slegtenhorst M., Dooijes D. (2012). Contractile dysfunction irrespective of the mutant protein in human hypertrophic cardiomyopathy with normal systolic function. Circ. Heart Fail..

[58] Sadayappan S., de Tombe P.P. (2012). Cardiac myosin binding protein-C: Redefining its structure and function. Biophys. Rev..

[59] Westerfield M. (2000). The Zebrafish Book. A Guide for the Laboratory Use of Zebrafish (Danio rerio).

[60] Kimmel C.B., Ballard W.W., Kimmel S.R., Ullmann B., Schilling T.F. (1995). Stages of embryonic development of the zebrafish. Dev. Dyn..

[61] Cornet C., Calzolari S., Miñana-Prieto R., Dyballa S., Van Doornmalen E., Rutjes H., Savy T., D’Amico D., Terriente J. (2017). ZeGlobalTox: An innovative approach to address organ drug toxicity using zebrafish. Int. J. Mol. Sci..

[62] Milan D.J., Jones I.L., Ellinor P., Macrae C.A. (2006). In vivo recording of adult zebrafish electrocardiogram and assessment of drug-induced QT prolongation. Am. J. Physiol. Circ. Physiol..

[63] Dhillon S.S., Dóró É., Magyary I., Egginton S., Sík A., Müller F. (2013). Optimisation of embryonic and larval ECG measurement in zebrafish for quantifying the effect of QT prolonging drugs. PLoS ONE.

[64] Yang J., Xu X. (2012). Immunostaining of dissected zebrafish embryonic heart. J. Vis. Exp..

[65] Da’As S.I., Coombs A.J., Balci T.B., Grondin C.A., Ferrando A.A., Berman J.N. (2012). The zebrafish reveals dependence of the mast cell lineage on Notch signaling in vivo. Blood.

[66] Huang W.-C., Hsieh Y.-S., Chen I.-H., Wang C.-H., Chang H.-W., Yang C.-C., Ku T.-H., Yeh S.-R., Chuang Y.-J. (2010). Combined use of MS-222 (tricaine) and isoflurane extends anesthesia time and minimizes cardiac rhythm side effects in adult zebrafish. Zebrafish.

[67] Lee L., Genge C.E., Cua M., Sheng X., Rayani K., Beg M.F., Sarunic M.V., Tibbits G.F. (2016). Functional assessment of cardiac responses of adult zebrafish (*Danio rerio*) to acute and chronic temperature change using high-resolution echocardiography. PLoS ONE.

