# Supplementary material for: Transcriptome Profile Identifies Actin as an Essential Regulator of Cardiac Myosin Binding Protein C3 Hypertrophic Cardiomyopathy in a Zebrafish Model
Source: Int J Mol Sci. 2022 Aug 9;23(16):8840. doi: 10.3390/ijms23168840 (PMC9408294; doi:10.3390/ijms23168840)

Supplemental videos A-E. TALEN *mybpc3* KO displayed ventricular phenotypes in the zebrafish model: representative heart videos at ventral views at 72 hours post-fertilization (hpf). (A) control showing the cardiac chambers(atrium (a) and ventricle (v)). (B-E) Zebrafish *mybpc3* mutant displayed ventricular phenotypes examined in different clutches. Zebrafish larvae were mounted into 3% methylcellulose and video recordings were taken using an Axio-Zoom V16 stereomicroscope equipped with an Image Source Camera (60 frames per second) at 100X magnification.

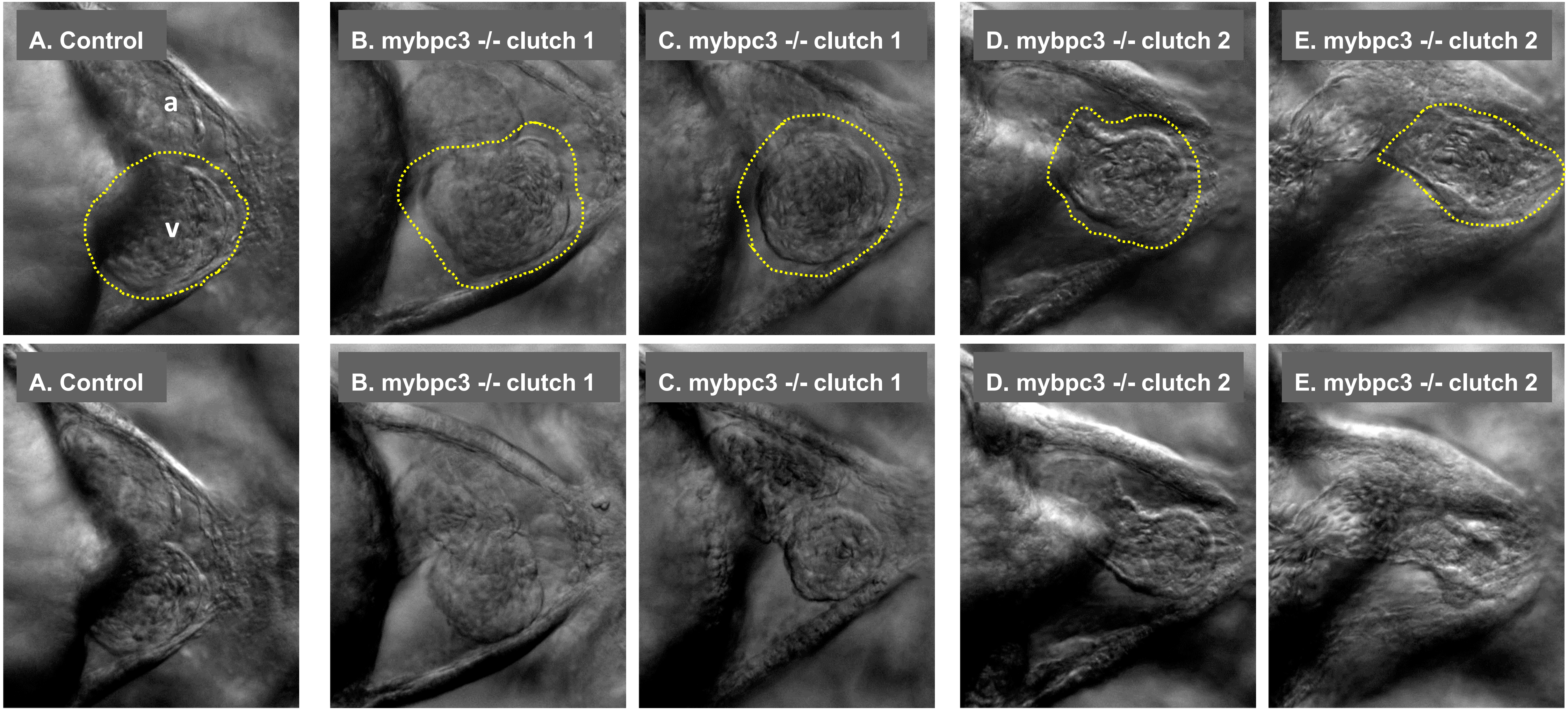

Supplemental videos F-I. TALEN *mybpc3* KO displayed ventricular phenotypes in the zebrafish model: representative heart videos at lateral views at 72 hours post-fertilization (hpf). (F) control. (G-I) Zebrafish *mybpc3* mutant displayed ventricular phenotypes examined in different clutches. Zebrafish larvae were mounted into 3% methylcellulose and video recordings were taken using Zeiss lumar 12 stereomicroscope equipped with an Image Source Camera (60 frames per second) at 120X magnification.

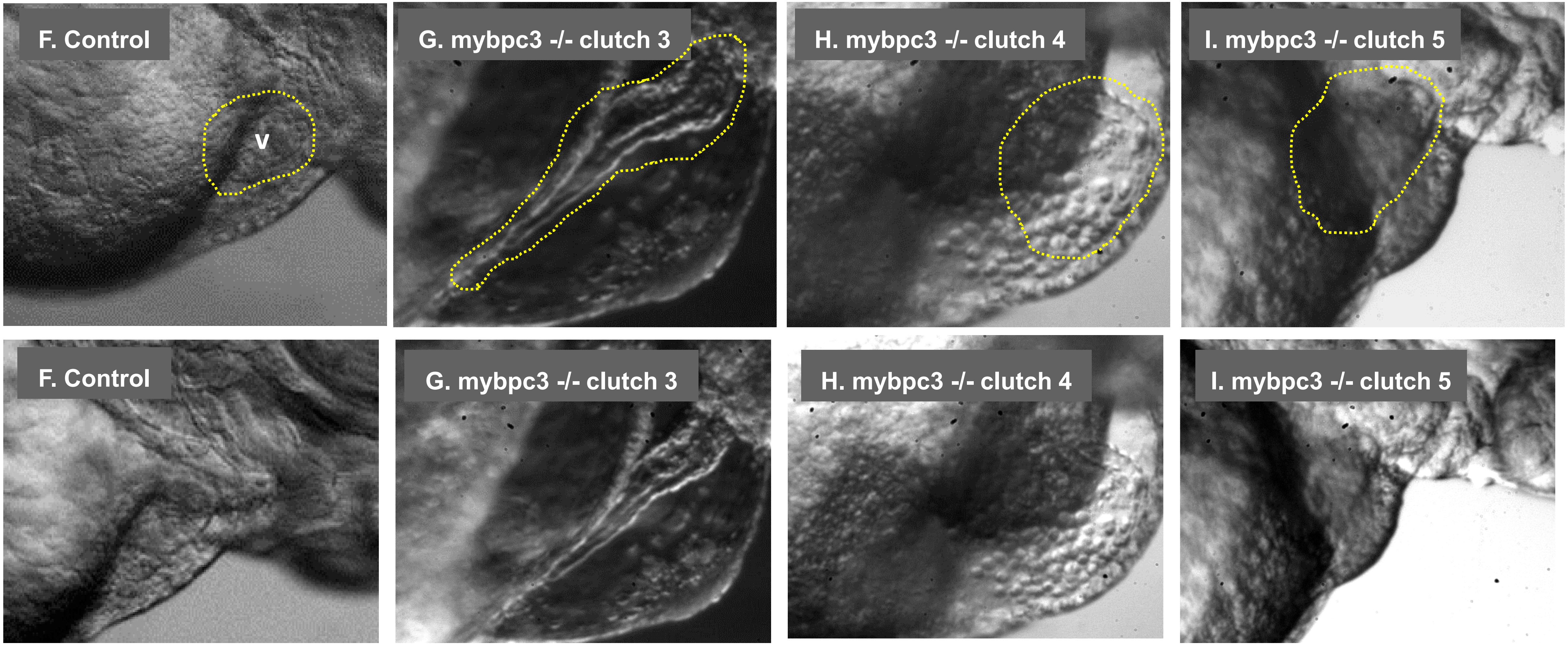

Supplement: Supplementary file 1 [file ijms-23-08840-s001.zip › MYPBC3 Supplemental videos.pdf]
